# Antimicrobial mechanism of semi-bionic extracts of three traditional medicinal plants—*Rheum palmatum L., Scutellaria baicalensis Georgi*, and *Houttuynia cordata Thunb*—That can be used as antibiotic alternatives

**DOI:** 10.3389/fvets.2022.1083223

**Published:** 2023-01-13

**Authors:** Hong Jiang, Zixia Bai, Ziheng Xu, Jian Sun, Hatungimana Françoise, Zuxiang Luan, Hongjun Wang

**Affiliations:** ^1^Institute of Animal Husbandry and Veterinary Medicine, Jinzhou Medical University, Jinzhou, Liaoning, China; ^2^Department of Pharmacy, Tianjin Baodi Hospital, Baodi Clinical College, Tianjin Medical University, Tianjin, China; ^3^School of Public Health and Management, Guang University of Chinese Medical, Nanning, Guangxi, China; ^4^Department of Animal Husbandry and Veterinary Medicine, Beijing Vocational College Agriculture, Beijing, China; ^5^College of International Education, Jinzhou Medical University, Jinzhou, Liaoning, China; ^6^Employment Department, Nanning Normal University, Nanning, Guangxi, China

**Keywords:** *Salmonella*, Chinese traditional medicinal plants, Dahuang Qinyu San, semi-bionic extract, antibacterial mechanism

## Abstract

The Chinese traditional medicinal plants *Rheum palmatum L., Scutellaria baicalensis Georgi*, and *Houttuynia cordata Thunb* in a ratio of 108:65:27 form a compound named Dahuang Qinyu San (DQS), which inhibits and kills *Escherichia coli* and *Salmonella* to a certain extent in fish and shrimp aquaculture environments. The active ingredients quercetin, emodin, baicalin, and aloe-emodin are obtained from the semi-biomimetic extract of DQS (SEDQS). However, the antibacterial mechanism of SEDQS against *Salmonella* is still unclear. This study used the microwell-plate method to determine the Minimum Inhibitory Concentration (MIC) of SEDQS against *Salmonella enteritidis* (*S. enteritidis*) isolated from geese. In addition, the effect of SEDQS on the growth curve, respiratory metabolic system, cell wall, soluble protein, and nucleic acid in bacterial liquid of *S. enteritidis* was detected by spectrophotometer and reagent kit. The effects of SEDQS on *S. enteritidis* DNA, binding gel blocking, virulence gene expression, and pathogenicity-related proteins were determined by gel electrophoresis, SDS–PAGE, and fluorescence quantitative PCR. The study found that a concentration of 1/4 MIC−2 MIC (2.27–18.2 mg/ml) SEDQS can significantly inhibit the normal growth of *S. enteritidis*, destroy the cell membrane structure of bacteria resulting in the leak of nucleic acid, protein, and other contents (*P* < 0.01). It also significantly inhibited the activities of succinate dehydrogenase (SDH) and malate dehydrogenase (MDH; *P* < 0.01) in a concentration-dependent manner. When the concentration of SEDQS was 1/2 MIC to 2 MIC (4.55–18.2 mg/ml), the expression levels of *gyrB, fimA, filC, spvR, Hcp*, and *vgrG* virulence genes (*P* < 0.01) all decreased by more than 31, 11, 18, 30, 34, and 21% respectively compared with the control group. SEDQS could significantly inhibit the expression of six virulence genes and play an important role in the pathogenicity of *the S. enteritidis* infected host. The SEDQS could exert antibacterial pharmacological effects by inhibiting the growth and metabolism of *S. enteritidis* and inhibiting the expression of major virulence factors. It has potential application value as an antibiotic alternative.

## Introduction

*Salmonella enteritidis* (*S. enteritidis*) exists in the digestive tract of healthy geese but it can also be found in humans, poultry, livestock, pets, rodents, wild animals, reptiles, and insects. It is often detected in meat, eggs, milk, animal products, and visceral infected animals. It is a kind of zoonotic pathogen and an important source of food-borne diseases causing high morbidity and mortality worldwide. About 70%−80% of global bacterial food poisoning cases are in China and 90% of them are caused by contaminated meat and other animal products (1, 2). Contact or consumption of contaminated food or water causes a series of diseases such as salmonellosis, also known as paratyphoid fever, chicken white diarrhea, septicemia, gastroenteritis, and other local tissue inflammations (3, 4).

In addition, sick and asymptomatic geese can be infection sources, which pollute the environment, affect other geese and further spread the disease. Furthermore, salmonellosis in geese is often mixed with other pathogenic bacteria, including *Escherichia coli*, and the pathogenicity and drug resistance of *Salmonella* in geese have continued to increase (5). This has serious detrimental effects on the economic benefits of the animal breeding industry, harms livestock production, and food safety, and affects the safety of public health at large. In recent decades, the use of antimicrobial agents has been considered the most important way to treat and control *Salmonella* infection. However, their toxicity and other side effects such as allergic reactions, double infections, bacterial resistance, and residues in animal products have gradually restricted their application (4). An alternative compound in Chinese medicines was discovered to have many pharmacological activities, such as clearing heat, detoxification, sterilization, and immune promotion, and has shown some promising results in the treatment of bacterial diseases (6, 7).

Current research and analysis methods of traditional Chinese medicine still use traditional solvent extraction methods lacking the metabolic process of drugs in the human gastrointestinal tract. The extraction of Chinese herbal medicine can be performed by semi-bionic extraction technology (SBE), which is a new type of traditional Chinese medicine preparation that is applied to the gastrointestinal tract by simulating the principles of oral administration and drug transport through the gastrointestinal tract from the perspective of biopharmaceuticals. There has been significant progress in the modernization of traditional Chinese medicinal powders, and the extraction processes follow the guidelines of traditional Chinese medicine use and the characteristics of the human gastrointestinal environment. In addition, SBE is a better choice and has different advantages when compared to the complex chemical components in a multi-herb prescription. It is a “perfect incorporation” of inheritance and innovation embodying the fundamental principles of traditional Chinese medicine. In addition, because organic solvents are not used in SBE (8), there is no residue of organic solvent in the extraction and the extraction temperature is relatively lower. This ensured that components that are sensitive to heat are not impacted. Moreover, SBE is in line with Chinese medicine theory, paying attention to pharmacodynamics response in the extraction process design. Recently, many researchers have investigated the possible utilization of the semi-bionic extract of some compounds in Chinese medicine to treat a few human diseases, and the results have been significant. For example, Wang et al. found that the semi-bionic extraction of the compound turmeric has potent protective effects on dextran sulfate sodium-induced acute enteritis in rats through its anti-inflammatory and antioxidant activities (9). Since 1995, several traditional Chinese medicines, and some Chinese herbal compound prescriptions have been studied, and all results indicated that the SBE method may replace the water extraction method (WE method), and the semi-bionic extraction alcohol precipitation method (SBAE method) may replace the water extraction and alcohol precipitation method (WAE method) (10). However, the SBE approach has not been applied in the extraction of traditional herbal medicine outside of China.

Previous studies have demonstrated the good antibacterial activity and potency of semi-bionic extracts from Chinese medicine compounds against pathogenic bacteria. Thus, traditional Chinese compound medicine (CCM) has become a far highly effective antimicrobial medicine in the development of a new semi-bionic anti-Salmonella drug, which is powerful with high antibacterial activity, safe, and reliable. In CCM, DQS is a classic formula commonly used in the Chinese Veterinary Pharmacopeia comprised of three kinds of Chinese herbs: *Rhubarb, Scutellaria baicalensis*, and *Houttuynia cordata*. It is used to clear away heat, detoxify, and treat fish and shrimp with gill rot disease (11). Therefore, DQS shows a certain inhibition and killing effect on *E. coli* and *Salmonella* in fish and shrimp aquaculture environments, but the mechanism of its action is still unclear.

In our previous study, we obtained the extract and content of quercetin, emodin, baicalin, and aloe-emodin, and using High-Performance Liquid Chromatography (HPLC, Japan HITACHI Corporation), we made a preliminary study on its composition and activity (12, 13). But its antibacterial mechanism has not been thoroughly explored. Based on the above analysis, the goal of this research is to investigate the mechanism of SEDQS against *S. enteritidis*.

## Materials and methods

### The growth curve of *S. enteritidis*

*Salmonella enteritidis* was isolated from a goose farm located in Jinzhou, China, and identified by Professor Tiezhong Zhou of Jinzhou Medical University.

A single colony of *S. enteritidis* was inoculated into the nutrient broth and cultured at 37°C for 18 h. The medium liquid was set as a blank control group. A spectrophotometer was used to measure the absorbance OD by ultraviolet-visible spectrophotometer (UV-1800, Shanghai Mepda Instrument Co., Ltd.) at 600 nm for 0, 2, 4, 6, 8, 10, 12, and 14 h, respectively, and the average of three repeated experiments was taken. The normal growth curve of *S. enteritidis* was plotted.

The liquid medium was inoculated with 10^4^ CFU/ml of *S. enteritidis* cultured to the logarithmic phase. An appropriate amount of drug was added to make final minimum inhibitory concentrations (MIC) of 2 MIC, MIC, 1/2 MIC, 1/4 MIC, and 1/8 MIC, respectively. A blank control group was set up (not adding the bacteria solution group) and cultured on a shaker at 37°C and 130 rpm for 5 min. A spectrophotometer was used to measure the absorbance OD at 600 nm for 0, 2, 4, 6, 8, 10, 12, and 14 h for each group, and take the average of 3 repeated experiments. The growth curve of *S. enteritidis* after the action of the SEDQS was plotted (with OD value as the ordinate, and time as the abscissa).

### Influence of SEDQS on the nucleic acid substance of *S. enteritidis*

#### Drug treatment

10^4^ CFU/ml of *S. enteritidis* cultured to the logarithmic phase was inoculated into a test tube containing a fresh medium. An appropriate amount of SEDQS was added to make the final concentration of 2MIC, MIC, 1/2 MIC, 1/4 MIC, 1/8 MIC, and 1/16 MIC. The medicines of each concentration without adding the bacteria were taken as the blank control group and cultured on a shaker at 37°C and 130 rpm.

#### Sample preparation

Two milliliter of each group of samples for 0, 2, 4, 6, 8, 10, and 12 h were taken, centrifuged for 10 min at 4,000 rpm, precipitate discarded, and the supernatant saved.

#### Detection of the sample

An ultraviolet spectrophotometer was used to measure the absorbance of DNA, RNA, and other macromolecular substances in the supernatant at a wavelength of 260 nm. The corresponding drug at each concentration was taken as the control group, and the average of three repeated experiments was taken. The curve of SEDQS's influence on the nucleic acid substance of *S. enteritidis* was plotted, with time as the abscissa and OD as the ordinate.

### Effect of SEDQS on the cell wall of *S. enteritidis*

The MIC was prepared as described previously (13). 10^4^ CFU/ml of *S. enteritidis* cultured to the logarithmic phase was inoculated into the liquid medium containing 2 MIC, MIC, 1/2 MIC, 1/4 MIC, and 1/8 MIC of SEDQS, and cultured at 37°C, 130 rpm on a shaker. Sterile water was used as a control group. The samples were centrifuged for 10 min at 5,000 rpm for 0, 2, 4, 6, 8, 10, and 12 h, respectively, and the supernatant was collected. The supernatant was added to a 96-well plate (Table 1).

**Table 1:** Determination of alkaline phosphatase.

	**Measuring hole**	**Standard hole**	**Control well**
Sample to be tested/μl	5		
0.1 mg/ml phenol standard/μl		5	
Double distilled water/μl			5
Diluent/μl	50	50	50
Matrix fluid/μl	50	50	50
**Incubate at 37**°**C for 15 min**			
Chromogenic agent/μl	150	150	150

The absorbance of each sample in the 96-well plate was measured at 520 nm with a microplate reader, which detected the change in absorbance at each time point over time and measured the absorbance three times in parallel to get the average value. The alkaline phosphatase (AKP) content was calculated according to the following formula.

Alkaline phosphatase (Kin's unit/100 ml) = [(measurement hole-blank hole)/(standard empty-blank hole)] × the concentration of phenol standard (0.02 mg/ml) × 100 ml.

### Effect of SEDQS on the respiratory and metabolic system of *S. enteritidis*

#### Drug treatment

10^4^ CFU/ml of *S. enteritidis* cultured to the logarithmic phase was inoculated into a fresh medium, so that the final concentration of SEDQS in the cultured medium was 2 MIC, MIC, 1/2 MIC, and 1/4 MIC. The sterile water was the blank control group, cultured on a shaker at 37°C and 130 rpm for 10 h. Three parallels were set for each group.

#### Sample preparation

The cultured bacterial solution was centrifuged at 5, 000 rpm for 10 min, the supernatant was discarded and the precipitate was retained. The treated samples were washed with 0.1 mol/L Tris-HCl (pH 7.4) buffer 2–3 times, and then an equal volume of lysozyme solution was added. After 15 min in a 37°C water bath, it was quickly transferred to an ice bath and treated with Tris-1 ml of SDS (pH 9.0) buffer before being centrifuged at 10,000 rpm for 20 min and the supernatant was collected for detection.

#### Determination of protein content (Bradford Protein Assay Kit P0006, Biyuntian Biotechnology Co., Ltd.)

The protein standard was diluted with phosphate-buffered saline (PBS) to make 6 different concentrations of 1.5, 1, 0.75, 0.5, 0.25, and 0.125 mg/ml. A 5 μl of each histone standard dilution solution was taken and added to the protein standard wells of the 96-well plate. A 5 μl of the sample was taken to be tested and added to the sample well. A 250 μl G250 staining solution was also added to each well. The absorbance of each well was measured at 570 nm with a microplate reader. According to the protein standard curve, the protein content in the sample was determined.

#### Determination of succinate dehydrogenase (Succinate Dehydrogenase Assay Kit A022, Nanjing Jiancheng Institute of Biotechnology) content

For 5 min, the prepared working solution was pre-warmed at 37°C. A volume of 100 μl of the sample to be tested was added into the test tube to which 2.6 ml of the working solution was pipetted and mixed immediately. The mixed solution was poured quickly into a cuvette and the color was compared at OD 600 nm with a visible spectrophotometer. The absorbance was read at 5 s and the absorbance was read again at 1 min 5 s to find the difference in absorbance twice. The binding protein content was calculated according to the formula below.

$$\begin{array}{l} \text{SDH}=\frac{\Delta OD\text{ value}÷0.01}{\text{reaction time }\left(\min\right)}\;\\ ÷\left(\text{sample amount x protein concentration of the sample to be tested}\right) \end{array}$$

#### The determination of malate dehydrogenase concentration (Malate Dehydrogenase Assay Kit A020-2, Nanjing Jiancheng Institute of Biotechnology)

The prepared working solution was pre-warmed at 37°C for 3 min. One hundred microliter of the sample to be tested was added into the test tube, and 1 ml of the working solution was taken and quickly flushed into the test tube and mixed immediately. The mixture was quickly poured into a colorimetric dish and the color was compared at 340 nm. The absorbance was read at 20 s and read again at 1 min 20 s. After calculating the difference in absorbance two times, the malate dehydrogenase (MDH) concentration of the binding protein content was calculated according to the formula below.

$$\begin{array}{l} \text{MDH }=\;\frac{\Delta OD}{3.1\;\times \;rection\;time\left(\min\right)}\\ \;\times \;\frac{\text{the total volume of the reaction liquid}}{\text{sampling amount }\times \text{ protein concentration of sample to be tested}} \end{array}$$

### SDS–PAGE method to detect the change of soluble protein content in bacterial liquid

#### Protein sample preparation

10^4^ CFU/ml of *S. enteritidis* cultured to the logarithmic growth phase was inoculated into a fresh medium. An appropriate amount of SEDQS was added to make the final concentrations of 2 MIC, MIC, 1/2 MIC, and 1/4 MIC. The sample was incubated on a shaker at 37°C at 130 rpm for 10 h. Sterile water was used as a control. The bacterial suspension collected from the control group and SEDQS water extract group was centrifuged, and the supernatant was discarded. The precipitate was diluted with PBS to make the absorbance 0.6. Then, the same volume of bacterial suspension was taken, centrifuged, the supernatant discarded, and the precipitate was suspended in a mixture of sample buffer and sterile water with a volume ratio of 1:4. The mixture was boiled in a water bath at 100°C for 10 min, then centrifuged at 4°C at 4,000 rpm, after which the precipitate was discarded. The supernatant was then retained for soluble protein. The preparation of separating gel and concentrated gel is shown in Table 2.

**Table 2:** Preparation of separating glue and concentrated glue.

**Reagent**	**10% Separating gel**	**5% Concentrated gel**
30%Acr-Bis/ml	3.3	0.5
1 M Tris, pH 8.8/ml	3.8	0.38
10% SDS/ml	0.1	0.03
10% ammonium persulfate/ml	0.1	0.03
TEMED/ml	0.004	0.003
Distilled water/ml	2.7	2.1
Total capacity/ml	10	3

#### Electrophoresis method

Before electrophoresis, two glass plates were cleaned, clamped with a clamp, filled with double distilled water, and let stand for 15 min. If no water flowed out, then the following experiment was carried out. An absorbent paper was used to absorb the remaining water in the glass plates. The prepared separating glue was mixed and quickly added to the gap between the two glass plates. The glue injection was stopped at a distance of 3 cm from the top. Then, isopropanol was added and placed at room temperature. Once the separation gel was polymerized after absorbing the isopropanol, slowly 5% concentrated gel solution was added to the top of the separation gel. The comb was inserted and left at room temperature. When the concentrated gel polymerization was completed, the glass plate was moved to the electrophoresis tank. The prepared electrophoresis buffer was poured into the tank, and the comb was slowly pulled out. A pipette was used to take 10 μl protein samples. The samples were added from the left side in order, after which the device was installed, and electrophoresis started at 220 V. When the band reached the separation gel, the voltage was adjusted to 90 V and the time was determined according to the marker. After the electrophoresis was completed, the gel was taken out, placed in the Coomassie Brilliant Blue dye solution, quickly stained, and decolorized with pure water until the band was visible. Finally, the gel imaging system was used to observe and take pictures. Protein electrophoresis bands were analyzed using Gel-Pro analyzer 4 software.

### Gel-blocking experiment of SEDQS and bacterial DNA binding

#### DNA extraction (bacterial genomic DNA extraction kit DP302, Beijing Tiangen Biochemical Technology Co., Ltd.)

10^7^ CFU/ml *S. enteritidis* cultured to a logarithmic growth phase was centrifuged at 5,000 rpm for 10 min. The DNA extract of the bacteria was collected using a bacterial DNA extraction kit. The ultra-micro UV spectrophotometer to measure OD260/280 = 1.82 > 1.8 met the requirements of this experiment. SEDQS for gradient dilution was used to prepare solutions of different concentrations.

#### Sample preparation

Five microliter of DNA extraction solution was accurately added to a sterilized 1.5 ml EP tube. Five microliter of different concentrations of SEDQS (2 MIC, MIC, 1/2 MIC, and 1/4 MIC, respectively) were also added, which had been serially diluted. The control group was replaced with sterile water.

#### Test samples (TC-412 PCR instrument, British TECHNE)

The test sample was incubated for 10 min at room temperature, 1% DNA agarose gel electrophoresis was performed, and pictures were taken with a gel imaging system to observe and record the results.

### Effect of SEDQS on bacteria genomic DNA content

#### Drug treatment

The *S. enteritidis* cultured to the logarithmic growth phase was centrifuged at 5,000 rpm for 10 min, rinsed with fresh medium, and diluted to a bacterial suspension of 10^6^ CFU/ml with the above-mentioned bacteria suspension. The liquid was divided into equal volumes. SEDQS was added to make the final concentrations of 2 MIC, MIC, 1/2 MIC, and 1/4 MIC, and sterile water was set as the control group.

The DNA at 37°C for 30, 60, 90, and 120 min was extracted and then centrifuged to collect the bacteria. Using a DNA extraction kit, the DNA of each group of bacteria was extracted. Test samples: 1% DNA agarose gel was used for electrophoresis, and pictures to observe and record the results were taken.

### Effects of SEDQS on the virulence gene of *S. enteritidis*

#### Extraction of strain RNA

The *S. enteritidis* grown to the logarithmic phase was inoculated into a fresh nutrient broth. An appropriate amount of SEDQS was added to make a concentration of 2 MIC, MIC, 1/2 MIC, and 1/4 MIC, and a drug-free control group was set up. After culturing on a shaker at 37°C and 130 rpm for 10 h, 1 ml of the bacterial suspension was taken, and the instructions for the bacterial total RNA rapid extraction kit provided by Shenggong Biological Company (Shanghai-China) were followed.

After 1 min at 8,000 rpm and 4°C, the medium was completely discarded, and 100 L of 400 g/ml lysozyme was added, shaken well, and enzymatically digested at room temperature for 5 min. After collecting the bacteria, 500 μl of DEPC-treated dd H_2_O was added to rinse the bacteria, centrifuged at 10,000 rpm for 1 min, and discarded the supernatant. Immediately, 900 μl of buffer Rlysis-B was added, shaken, mixed well, and left at room temperature for 3 min. A 200 μl of chloroform was added and thoroughly mixed. The mixture was centrifuged at 12,000 rpm at 4°C for 5 min and the supernatant was obtained. A 1/3 volume of absolute ethanol was added to the supernatant, mixed well, placed at room temperature for 3 min, centrifuged at 12,000 rpm at 4°C for 5 min, and the supernatant was discarded carefully. The pellet was washed with 700 μl of 75% ethanol (prepared with DEPC-treated dd H_2_O), centrifuged at 12,000 rpm at 4°C for 3 min, and the supernatant was discarded carefully. The above step was repeated once. It was inverted at room temperature for 10 min, and 50 μl of DEPC-treated dd H_2_O was added to dissolve the precipitate and used immediately. A spectrophotometer UV-1800 was used to detect the purity of RNA.

#### Synthesis of cDNA

Five microliter of total RNA, 1 μl of Oligo (dT) 18 primer, and 6 μl of water (nuclease-free) were added into the PCR tube. The above system was put in a 65°C water bath for 5 min. Reverse transcription reaction system 5 × Reaction Buffer 4 μl, Ribolock RNase Inhibitor (20 U/μl) 1 μl, 10 mM dNTP Mix 2 μl, and RevenAid M-MuLV RT (200 U/μl) 1 μl were added to the reference kit. Reaction conditions were: 42°C, 60 min; 70°C, 5 min.

#### Primer design

The Tiangen Biological Company (Beijing-Chana) synthesized the primers referenced in the literature. The primer sequence and fragment size are shown in Table 3.

**Table 3:** Primer sequence used for real-time RT-PCR.

**Gene**	**Primer sequence (5^′^-3^′^)**	**Product size bp**	**References**
*dnaE* (internal reference)	F: GATTGAGCGTTATGTCGGAGGC	80	(14)
	R: GCCCCGCAGCCGTGAT		
*gyrB*	F: GTCGAATTCTTATGACTCCTCC	111	(15)
	R: CGTCGATAGCGTTATCTACC		
*fimA*	F: CCT TTC TCC ATC GTC CTG AA	85	(14)
	R: TGG TGT TAT CTG CCT GACCA		
*filC*	F: GCAGATGACGGTACATCCAA	180	(16)
	R: CCAGATCAGGCTGTGCTTTA		
*spvR*	F: CAG GTT CCT TCA GTA TCG CA	310	(17)
	R: TTT GGC CGG AAA TGG TCA GT		
*Hcp*	F: AAGTCCACTCAACAGCAGCA	100	(14)
	R: TTT GGC CGG AAA TGG TCA GT		
*vgrG*	F: GCGGATCCATGTCCTTAAAAGGTCTTCG	450	(15)
	R: GTGCTCGAGTCCGTTCAGATGAATATC		

### Detection of changes in virulence genes by RT-PCR

2× Super Real PreMix Plus (stored at −20°C), 50× ROX Reference Dye, template, primers, and RNase-Free dd H_2_O were dissolved. The RT-PCR reaction solution was prepared in an icebox. See Table 4 for the reaction system. The tube cap was covered and shaken gently to mix. Fluorescence quantitative PCR (ABI 7500 Fluorescence quantitative PCR, American ABI) amplification was carried out with a two-step method, the reaction conditions were 95°C pre-denaturation for 15 min; 95°C 10 s, 60°C 30 s, 40 cycles.

**Table 4:** Fluorescence quantitative PCR reaction system.

**Element**	**Dosage/μl**
2 × SuperReal PreMix Plus	10
Primer (upstream)	0.6
Primer (downstream)	0.6
cDNA template	1
50× ROX reference dye	0.5
RNase-Free ddH_2_O	7.3
Total	20

### Data processing and statistical analysis

In this research, all experiments were repeated three times. The data obtained were analyzed by stepwise regression analysis and one-way analysis of variance using SPSS 25.0 software, expressed as mean ± standard deviation (*X* ± SD). The criteria for determining statistical results were as follows: *P* < 0.01 meant the data difference was extremely significant; 0.01 < *P* < 0.05 meant the data difference was significant; *P* > 0.05 meant the data difference was not significant.

## Results

### The normal growth curve of *S. enteritidis*

**Figure 1:**
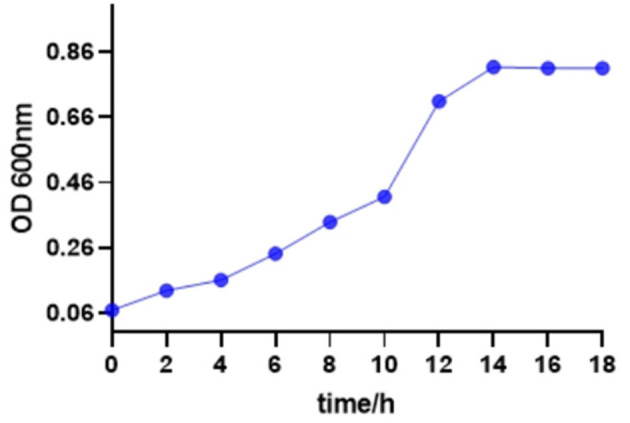
Growth curve of *Salmonella enteritidis*.

As shown in Figure 1, the bacterial growth of *S. enteritidis* was relatively slow until 8 h. After 10 h, the growth rate of *S. enteritidis* increased rapidly, indicating the logarithmic phase of bacteria growth, and after 14 h, the stable phase was attained. That showed favorable conditions for bacteria growth.

### The growth curve of *S. enteritidis* under the action of the SEDQS

**Figure 2:**
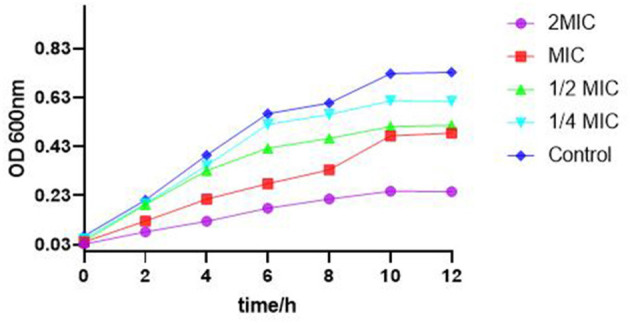
Effect of SEDQS on the growth curve of *Salmonella enteritidis*.

As shown in Figure 2, the bacteria in the control group multiplied rapidly before 6 h and entered the stable phase of the growth curve after 10 h. Compared with the control group, when the SEDQS concentration increased from 1/4 MIC, 1/2 MIC, MIC, and 2MIC, the bacteria growth decreased, respectively. At 2 MIC and MIC, the growth of bacteria did not show an obvious logarithmic phase. This indicated a significant inhibition of the bacteria by SEDQS.

### Effect of SEDQS on the nucleic acid substance of *S. enteritidis*

**Figure 3:**
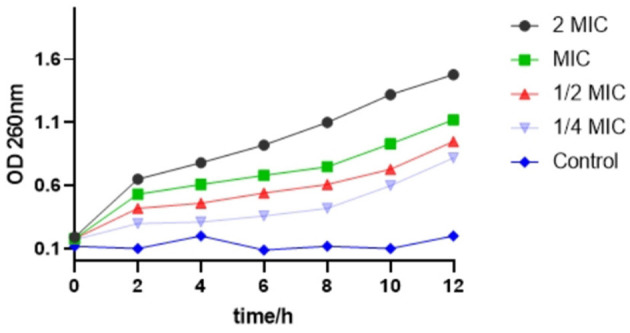
The change about OD of nucleic acid substance from *Salmonella enteritidis* affected by SEDQS.

As shown in Figure 3, with an increase in time, the OD value of the bacterial suspension added with SEDQS also increased, indicating the exudation of bacterial macromolecules into the liquid. This implied that the bacterial cell membrane permeability increased with an increase in drug concentration. The cell membrane is one of the protective barriers against bacteria. Consequently, when the cell membrane is damaged, the permeability of the cell membrane changes, which may cause DNA, RNA, and other macromolecules that, cannot penetrate the cell membrane under normal conditions to leak through the cell membrane into the culture medium that resulted in the increasing trend of an absorbance value of the culture medium at 260 nm.

The OD value in the bacterial solution treated with 2 MIC solution was significantly higher than that of the bacterial solution treated with MIC, 1/2 MIC, and 1/4 MIC solution, respectively, and the difference was extremely significant (*P* < 0.01). In the control group, the cell membrane was intact, and the OD value did not change significantly.

### Activity of SEDQS on the cell wall of *S. enteritidis*

**Figure 4:**
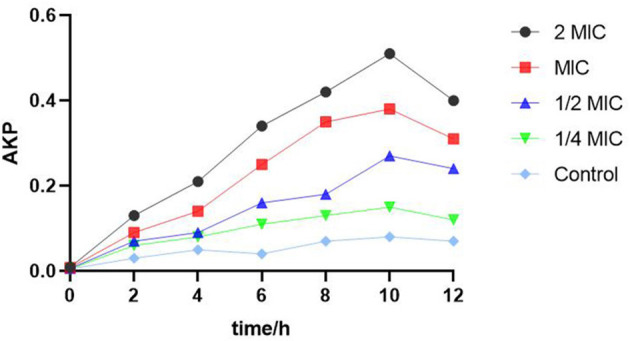
The AKP content of *Salmonella enteritidis* treated with SEDQS.

Figure 4 shows that the content of extracellular AKP in *S. enteritidis* after the treatment of SEDQS was significantly higher than that in the control group, and it was proportional to the concentration of the extract, indicating that SEDQS had a certain destructive effect on the cell wall of bacteria.

### Effect of SEDQS on the respiratory and metabolic system of *S. enteritidis*

#### Standard curve of protein content

**Figure 5:**
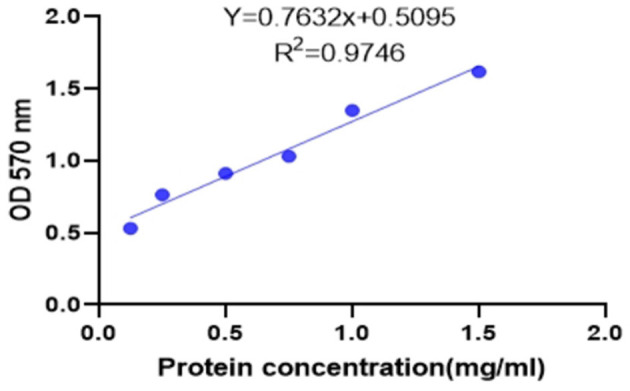
Protein concentration standard curve.

As shown in Figure 5, linear regression was performed with the absorbance (Y, OD570 nm) as the ordinate and the protein concentration (*X*, mg/ml) as the abscissa. The regression equation of the protein concentration was obtained as *Y* = 0.7632 *X* + 0.5095, *r*^2^ = 0.9746 (*n* = 6), indicating that the protein concentration had a good linear relationship with the absorbance at 570 nm in the range of 0.125–1.5 mg/ml. The protein concentration of the sample can be calculated from this formula.

#### SDH activity detection

The experimental results in Table 5 show that the activity of succinate dehydrogenase (SDH) in the 1/4 MIC, 1/2 MIC, MIC, and 2 MIC groups was reduced by 26.32, 41.59, 67.19, and 76.81% respectively, compared with the control group (*P* < 0.01). The results indicated that SEDQS at a dose of 2.27–18.2 mg/ml could inhibit the SDH activity of *S. enteritidis*.

**Table 5:** Effect on SDH of bacteria treated with SEDQS.

**Group**	**ΔA_570_**	**Protein content (mg/ml)**	**SDH activity (U/mgprot)**
Control	0.027 ± 0.008	0.803 ± 0.091	32.978 ± 0.788
1/4 MIC	0.023 ± 0.011	0.717 ± 0.106	24.296 ± 0.556^**^
1/2 MIC	0.012 ± 0.003	0.65 ± 0.079	19.262 ± 0951^**^
MIC	0.008 ± 0.002	0.592 ± 0.057	10.818 ± 0.689^**^
2 MIC	0.003 ± 0.001	0.519 ± 0.029	7.647 ± 1.127^**^

Compared with the control group,

** means a significant difference at P < 0.01.

The one-way ANOVA results showed that there were statistically significant differences between 2 MIC, MIC, 1/2 MIC, and 1/4 MIC of SEDQS (P < 0.01). The activity of SDH decreases according to the concentration of the extract.

#### MDH activity detection

The experimental results in Table 6 show the activity of MDH in the 1/4 MIC, 1/2 MIC, MIC, and 2 MIC groups were reduced by 16.63, 29.85, 51.99, and 74.33% respectively compared with the control group (*P* < 0.01). The results indicated that SEDQS at a dose of 2.27–18.2 mg/ml could inhibit the MDH activity of *S. enteritidis*.

**Table 6:** Effect on MDH of bacteria treated with SEDQS.

**Group**	**ΔA_340_**	**Protein content (mg/ml)**	**MDH activity (U/mgprot)**
Control	0.057 ± 0.019	0.803 ± 0.091	43.013 ± 0.650
1/4 MIC	0.041 ± 0.014	0.717 ± 0.106	35.856 ± 0.586^**^
1/2 MIC	0.03 ± 0.007	0.65 ± 0.079	30.172 ± 0.281^**^
MIC	0.024 ± 0.004	0.592 ± 0.057	20.649 ± 0.582^**^
2 MIC	0.008 ± 0.001	0.519 ± 0.029	11.039 ± 0.772^**^

Compared with the control group,

** means a significant difference at P < 0.01.

The one-way ANOVA results showed that there were statistically significant differences between 2 MIC, MIC, 1/2 MIC, and 1/4 MIC of SEDQS (P < 0.01). The activity of MDH decreases according to the concentration of the extract.

### The effect of SEDQS on the soluble protein in *S. enteritidis*

**Figure 6:**
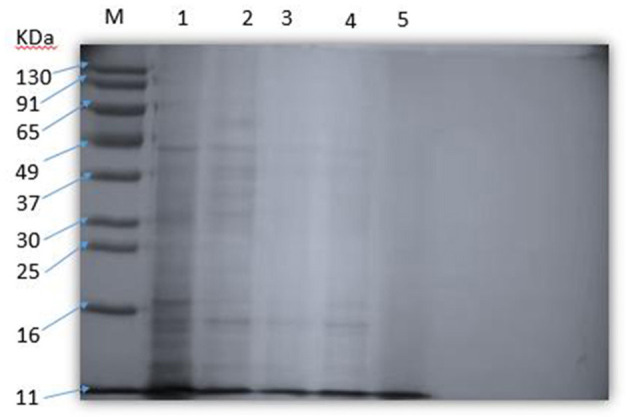
The soluble protein of *Salmonella enteritidis* by SEDQS. M: Marker; 1: control group; 2: 1/4 MIC treatment group, 3: 1/2 MI treatment group C; 4: MIC treatment group; 5: 2 MIC treatment group.

The effect of SEDQS on the content of soluble protein in *S. enteritidis* is shown in Figure 6. The protein analysis software Gel-Pro Analyzer quantitatively analyzed the SDS–PAGE protein bands. The results showed that with increasing the drug concentration, the soluble protein content decreased or even disappeared compared with the control group. It is suggested that SEDQS inhibits the expression of these proteins and their synthesis by *S. enteritidis*.

### Gel-blocking experiment of SEDQS and bacterial DNA binding

**Figure 7:**
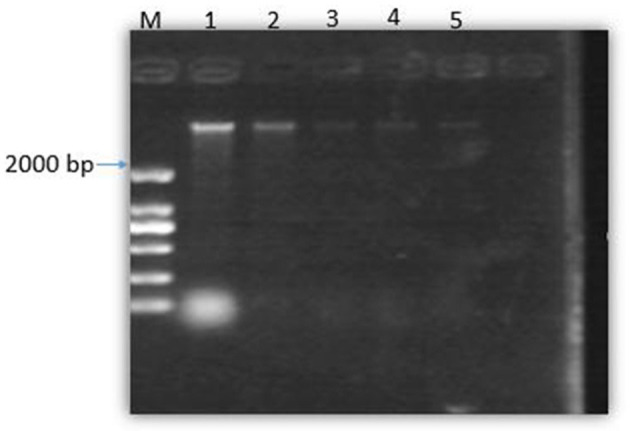
Gel of bacterial DNA binding to SEDQS. M: Marker, 1: DNA untreated; 2: DNA treated with 1/4 MIC, 3: DNA treated with 1/2 MIC, 4: DNA treated with MIC, 5: DNA treated with 2 MIC.

Figure 7 shows that with the increase in the concentration of SEDQS, the brightness of the DNA bands decreased progressively compared to the control. This indicates that SEDQS can be tightly combined with bacterial genomic DNA, resulting in mobility changes. The effect of the extract on bacterial genomic DNA shows that SEDQS has a strong interaction with genomic DNA.

### Effect of SEDQS on bacteria genomic DNA content

**Figure 8:**
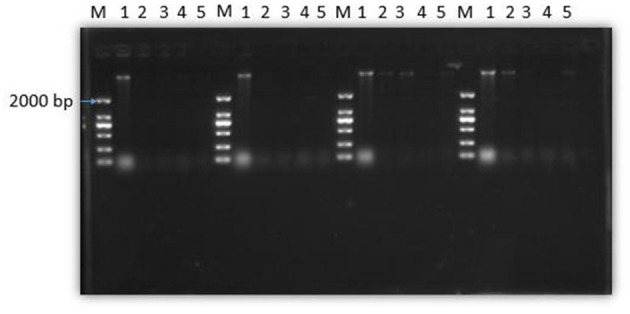
Agarose gel electrophoresis of DNA from bacterial cells treated by SEDQS. M: Mark; 1: untreated group; 2: 1/4 MIC, 3: 1/2 MIC, 4: MIC, 5: 2 MIC treated group after 30, 60, 90, and 120 min.

Figure 8 shows that the total amount of genomic DNA in the experimental group was significantly less than that in the control group after treatment with SEDQS at a concentration of 2 MIC, MIC, 1/2 MIC, and 1/4 MIC compared with the control group. With the prolongation of the treatment time of SEDQS, the brightness of the genomic DNA bands in the experimental group gradually weakened, indicating that the synthesis of bacterial DNA was decreasing, and it indicated that the synthesis of genomic DNA in SEDQS-acting bacteria was a continuous process.

### Effects of SEDQS on virulence genes of *S. enteritidis*

**Figure 9:**
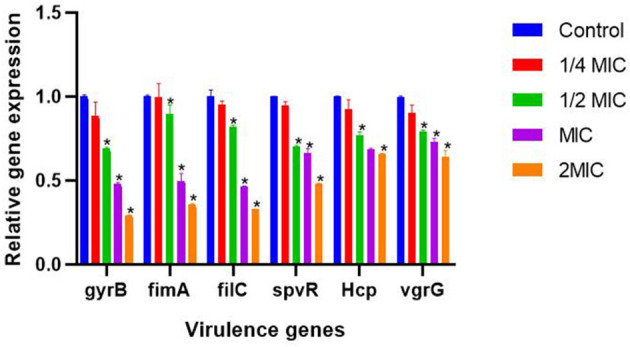
The effect of 1/4 MIC to 2 MIC SEDQ concentration on the expression of six virulence genes of *Salmonella enteritidis*. Compared with control group, * showed significant difference at *P* < 0.01.

As shown in Figure 9, the results showed that different concentrations of SEDQS significantly inhibited the six virulence genes of *Salmonella, gyrB, fimA, fliC, spvR, Hcp*, and *vgrG* (*P* < 0.01) compared with the control group.

SEDQS has the most obvious inhibitory effect on the expression of *gyrB, fimA, filC, spvR, Hcp*, and *vgrG* genes at 2 MIC (18.2 mg/ml), MIC (9.10 mg/ml), and 1/2 MIC (4.55 mg/ml), and their expression levels decreased by more than 31, 11, 18, 30, 34, and 21% respectively, compared with the control group. However, there was no significant effect at 1/4 MIC (2.27 mg/ml) of SEDQS on the expression of all virulence genes (*P* > 0.05). The different concentrations of SEDQS can inhibit the expression of virulence genes in *Salmonella*.

## Discussion

The antibacterial mechanism of traditional Chinese medicine is very complex because it contains multiple components. Traditional Chinese medicine often exerts antibacterial effects through multiple mechanisms, especially through compound traditional Chinese medicine (18). Currently, most traditional Chinese medicines can inactivate microbial organisms by destroying the cell wall structure, inhibiting nucleic acid transcription and replication, denaturing microbial proteins, interfering with their growth and reproduction, changing the permeability of cell membranes, interfering with the microbial enzyme systems, and destroying their normal metabolism and other pathways to exert a bacteriostatic effect (19).

The growth curve represents the proliferation of bacteria in the culture medium. To obtain and utilize probiotics, we can adjust the conditions of a bacterial culture by referring to the growth curve, and then adjust the growth and reproduction of bacteria. As a result, the bacterial growth curve has a guiding significance in scientific research and production practice (20). Bacterial growth is generally divided into four phases, namely, the lag phase, logarithmic growth phase (also known as the exponential), stable growth phase, and decay growth phase, which are reflected in the growth curve of the bacteria (21). The number of viable bacteria in the logarithmic phase increases logarithmically, and the bacterial morphology, staining, and physiological activity were typical and sensitive to the effects of external environmental factors. This phase is an ideal stage to study bacterial biological characteristics and drug sensitivity tests. However, the reproduction rate of bacteria in the stable period gradually decreases, after which the total number of bacteria tends to be flat. At this stage, bacterial reproduction and apoptosis gradually reach a balance, but their morphological structure, coloration, and biological properties have a chance to change and produce specific metabolites, such as spores, endotoxins, and exotoxins (22). The results of this study showed that the bacterial solution treated with different concentrations of SEDQS maintained a slow-growth state, indicating that SEDQS could significantly inhibit the reproduction and growth of *Salmonella* in a dose-dependent manner.

The macromolecules of a bacterial cell, including nucleic acids and proteins, which reside throughout the interior of the cell and cytoplasm are the key structural components. Severe damage resulted in the leakage of these macromolecules and the death of the cells (19, 21). In this study, the changes of DNA and RNA in the supernatant of SEDQS treated with *S. enteritidis* were detected by a spectrophotometer to reflect the degree of damage to bacterial nucleic acid. The results showed that the absorbance of each experimental group at OD_260_ was greater than that of the control group at different time points, and the difference was statistically significant. The leakage of nucleic acids and proteins could cause a disorder of function in the synthesis of proteins and DNA materials and inhibit bacterial growth.

The cell wall is one of the important components of bacteria. The main function of the cell wall is to maintain the inherent shape of the cell, which has a certain protective effect on the cell and is a barrier with unique biological significance. When the integrity of the cell wall of the bacteria is destroyed, the function of the permeability barrier was lost, which caused the leakage of intracellular components and led to the expansion and deformation of the bacteria, which finally lyse and die. Previous studies have reported similar results (22). By measuring the content of alkaline phosphate (AKP), we evaluated the integrity of the bacterial cell wall. In bacteria, AKP mainly exists between the cell wall and the cell membrane, and it can be leaked extracellularly by increasing the permeability of the bacterial cell wall. The values of AKP can reflect the integrity of the bacterial cell wall in an indirect way as reported in previous studies (19, 23). Our findings revealed that the content of AKP changed significantly after treatment with SEDQS in the range of 2.27 to 18.2 mg/ml, indicating that SEDQS destroyed the cell wall of the bacteria.

Respiratory metabolism is an important manifestation of the normal life activities of living organisms, mainly the oxidation of carbohydrates and lipids. Some drugs and bacteriostatic agents can affect bacteria's normal growth and reproduction by inhibiting their respiratory metabolism (24–26). SDH and MDH are the key enzymes of the tricarboxylic acid cycle (TCA) and play an important role in cellular energy metabolism. Changes can reflect the level of energy metabolism in cells (27–29). Our results show that SEDQS could inhibit the activities of SDH and MDH, indicating that the extract can inhibit the reproduction and growth of *S. enteritidis* by destroying the respiratory system.

Proteins are the material basis of life and are involved in various forms of activities. Many kinds of bacteriostatic agents and drugs can coagulate, denature, or reduce the synthesis of bacterial proteins, especially reducing a certain protease with catalytic function, thereby inhibiting the growth and reproduction of bacterial cells (30). In this study, SDS–PAGE electrophoresis band proteins were changed when bacteria were treated with SEDQS at 2 MIC, MIC, 1/2 MIC, and 1/4 MIC concentrations. The results showed that the protein expression of the bacteria decreased with drug concentration compared with the untreated group, which illustrated that there was a certain suppression of the normal metabolism of proteins. In brief, the bacteriostatic function of SEDQS showed inhibitory activity on *S. enteritidis* by decreasing the expression of the bacterial proteins.

Compared with the control group, the expression of DNA in the experimental group of bacterial cells treated at different times was significantly lower than that in the control group. The reason was that with the long-term treatment of bacterial cells, the bacteria continued to suffer until it died, and the amount of DNA synthesis was continuously reduced, which meant that it was a continuous process. These two experiments show that SEDQS can affect the expression and synthesis of DNA in bacteria to achieve the bacteriostatic effect. At the same time, it is also confirmed that the pathogenic microorganisms' DNA is one of the targets of antibacterial drugs.

The pathogenicity of bacteria is mainly related to its virulence genes. To accomplish host infection, *Salmonella* uses specific virulence factors to facilitate their adhesion, invasion, and multiplication in host cells and overcome host antimicrobial defenses. These virulence genes are mainly included in secretion systems, pathogenicity islands, virulence plasmids, flagella, and fimbriae (31–33). Among them, six virulence genes, such as *gyrB, fimA, fliC, spvR, Hcp*, and *vgrG*, were selected in this paper to detect the effect of SEDQS on the virulence genes of *S. enteritidis*. In the results of this experiment, SEDQS significantly inhibited the expression of *gyrB, fimA, filC, spvR, Hcp*, and *vgrG* virulence genes carried by *Salmonella*, but SEDQS at a concentration of 1/4 MIC (2.27 mg/ml) showed no significant effect on the relative expression of all genes.

The *gyrB* gene is a subunit of the DNA gyrase that maintains the topology of DNA by introducing negative supercoils using energy generated by ATP hydrolysis. This protein has been used as a phylogenetic marker for several genera closely related to Flavobacterium (34). The *fimA* gene is the gene encoding type I fimbriae of *Salmonella*, which plays a role in mediating bacterial adherence to eukaryotic cells, a critical step in the successful colonization and pathogenesis of *Salmonella* (35). The *fliC* gene is a flagellin protein that displays a distinct motility behavior on host cell surfaces and a competitive advantage in colonizing the intestinal epithelia in most *Salmonella* strains. It also plays a well-documented role in innate immunity and as a dominant antigen of the adaptive immune response. Importantly, flagella have also been reported to function as adhesins (36). *SpvR* is the first gene transcribed in the *Salmonella* plasmid virulence (*spv*) operon, which can regulate the expression of the other four genes, such as *spvA, spvB, spvC*, and *spvD*. These genes may increase the growth rate of *Salmonella* in host cells and affect the interaction of *Salmonellae* with the host immune system (37). *Hcp* and *vgrG*, both encoded by the type 6 secretion system (T6SS), correspond to the genetic characterization of Pathogenicity Island 6 (SPI-6). Translocation of *vgrG* and *Hcp* into the host cytoplasm influenced bacterial motility, protease production, and biofilm formation (38).

The changes in these six virulence genes were detected by RT-PCR, and the results showed that their expressions showed a downward trend after the bacteria were treated with SEDQS. SEDQS can reduce damage to the body by regulating the expression of virulence genes.

## Conclusions

In this study we reported that SEDQS has a MIC of 2.27–18.2 mg/ml against *S. enteritidis*, which can significantly inhibit bacterial growth and reproduction, destroy bacterial cell membrane structure, leak nucleic acids, and proteins, and reduce SDH and MDH activity. In addition, 4.55–18.2 mg/ml of SEDQS can significantly inhibit the expression of *gyrB, fimA, fliC, spvR, Hcp*, and *vgrG* virulence genes of *S. enteritidis*. The SEDQS could exert antibacterial pharmacological effects by inhibiting the growth and metabolism of *S. enteritidis* and inhibiting the expression of major virulence factors. It has potential application value as an antibiotic alternative.

## Data availability statement

The original contributions presented in the study are included in the article/supplementary material, further inquiries can be directed to the corresponding authors.

## Author contributions

Methodology and writing—original draft preparation: HJ, ZB, ZX, and HF. Validation: JS and HF. Software: HJ, HF, and ZL. Resources and formal analysis: HF and HW. Supervision, project administration, and writing—review and editing: ZX and HW. All authors have read and agreed to the published version of the manuscript.
